# Suggestion or Suggestive Therapeutics in the Practice of Medicine

**Published:** 1906-06

**Authors:** G. T. Kesner

**Affiliations:** Decatur, Ga.


### SUGGESTION OR SUGGESTIVE THERAPEUTICS IN THE PRACTICE OF MEDICINE.

By G. T. KESNER, M.D.,

DECATUR, GA.

I am certainly glad to have an opportunity to write a few lines on a subject that I think is quite an important one to the medical profession. It is the subject of Suggestion as a Therapeutic Agent in the Practice of Medicine.

We all use it to a certain extent. Some use it more and some use it less. Some use it knowingly and some use it unknowingly. Some physicians make it a study, while others look upon it as quackery.

The use of suggestion is not new. It has been used as a therapeutic measure from the earliest times down to the present, and its history through its various phases is quite an interesting study. The ancient priests used pure suggestion with more or less impressive incantatious ceremony to increase the faith of the people at that time.

It is the sheet anchor of the Christian Scientist, the armor-bearer of the magnetic healer, and the main prop of the faith doctor, mind curer, Osteopathic workers, and many others. All of these sects use suggestion in various ways and forms. Some of them use the hypnotic stroke in addition to suggestion. Some give credit principally to massage, claiming thereby the adjusting of the parts not in opposition. These workers continually and eternally keep telling their patients what they desire them to do and believe. The success which crowns their efforts in a large number of cases can not be denied or refuted. That they fail in a large number of cases can be proven, and therefore must be admitted. One of the causes of these failures is they often promise too much, or undertake to cure those diseases that are not curable by suggestion alone. Suggestion alone will only cure a limited number of cases. By closely observing their success or failure we can much better use suggestion to advantage.

I will admit that this is somewhat a sceptical subject to quite a number, but I believe that the time is coming, and, in fact, is nearly here, when suggestion will be used much more than at present. All are susceptible to its influence. A doctor can often make many a one feel badly by telling him that he looks sick.

My little children—ages eight and ten years—can be in bed at night talking with the greatest glee, when I can say to them, "Good-night; let's all go to sleep," and most of the time, in from three to five minutes, they will be sound asleep. The simple suggestion of sleep is all that is necessary in a great number of times.

Suggestion should be used in nearly, if not every case, in which we come in contact. In most cases we suggest the effect we wish the drug to have. It is a wonderful help. Thus, when a patient is in much pain, state to him that he will be easy in a very short time. And keep on suggesting at short intervals that he is not suffering quite so much, and that he is about easy, etc. Of course, I mean for you to give him the drugs indicated in his case in addition to the suggestion. If you say nothing in regard to the effect of your drugs, your patient will be slow in saying he is well, or even better. Nine times out of ten he will soon say he is feeling better, if you will merely suggest his improved condition.

In every case we should be positive. I often say to my patient, after thoroughly examining him, "You are quite sick, but I will soon have you feeling like a new fellow," or, "If you will take this medicine as directed, you will soon see a big change for the better." You might mention some cases just like his that you cured so quickly, etc. Never say that "I *hope* this medicine will do you good," or "If you are not better when I return I will put you on something else." The probability is he will not expect benefit, neither will he realize it. If you fail to relieve them (as you will now and then), you can say: "Well, it fails in about one time in five hundred, and *your* case happens to be one of the failures." No harm done, you see.

A little deception on our part is, I believe, excusable, *provided* it is used with no intention whatever to injure, but to benefit our patients. (I trust that no one will ever quote part of the above sentence without quoting all of it). If your patient believes and really *does have* some serious disease, it is not always best to tell him so. Neither is it best to tell him that nothing is the matter. You might suggest something like this: "You have a little heart disease and other troubles, but I can easily remedy that." (Of course, whatever disease he thought he had.)

In a case of military tuberculosis, or any disease where it would soon prove fatal, I would not deceive them in regard to their true condition. A falsehood is sometimes justifiable, but a lie, never.

We often admit what we know is not true, and it is sometimes absolutely necessary in the treatment of the case. Well do I remember a case I had a few years ago while practicing down in Southeast Georgia. A white woman about forty years of age was carrying wood, when she saw a little bug on her shoulder and, according to her story, it ran into her ear. I was sent for in haste. She felt it digging into her ear continually and causing her a great deal of pain. I at once had her seated in a chair near the window and proceeded to hunt for "Mr. Bug." I spread open the ear with a pair of long tweezers, but saw no bug. Her husband was standing near me watching my every movement. I told her to be perfectly still. I removed the tweezers rather quickly and gave them a little flip as if to throw the bug out of the window onto the woodshelf, with a remark something like this: "The infernal thing will never get into any one else's ear." That remark was sufficient. The old man looked for that bug on the shelf for two or three minutes, and the old lady had no more scratching in her ear. Suppose that I had told her that there was no bug in her ear? Why, of course, some other doctor would have been called. She was so positive that it was in her ear that I considered my treatment the best for her. If I had been called to see if a bug was in her ear, I certainly would have said there was not. But the case was quite different. That remark I made to them goes in the category of suggestion.

Don't think that I use suggestion to the exclusion of the necessary medicinal or surgical treatment. Far from it. While administering the drugs I give every possible assurance that they will soon be better.

Some time ago I was called to a case of pernicious malarial fever. Those of you who have practiced in malarial districts can probably better appreciate the following report of case. The patient—a girl sixteen or eighteen years of age—had had a paroxysm just before they sent for me. When I got there she was comparatively easy. The circulation was only fairly good. Temperature was only slightly elevated. Now and then she gave a long breath and a sigh, and had a few other symptoms that I did not like. I gave her a heart stimulant and five grains of quinine. As it was now noon, and I was a good ways from home, we went in to dinner. As I left the room I informed the girl's parents that she was dangerously sick, and that she had symptoms that were serious, and that she was likely to have other paroxysms. As we finished eating she had another paroxysm in which she very nearly died. However, she did not lose consciousness. Her feet and hands grew cold, her pulse was not perceptible, she said that she was dying, she bid her friends and relatives good-by and requested them to pray for her. Her circulation was still getting weaker, the surface of her body was getting colder, and complete congestion was very near at hand. Here was a case for medicinal treatment and *suggestion.* Of course, we did not wait till this stage of the disease above mentioned before beginning the treatment. I gave her a puncture of strychnine sulphate 1.30 gr., and morph. mulph. 1-4 gr., and Atropu. sulph. 1-50 gr., and also a hot toddy. We also used hot applications of mustard, etc., to hands and feet, and most parts of the body. In addition to this I assured her with all positiveness that she was not going to die. That her pulse was strong, even better than mine while in health; that her heart was beating strong and regular; that her feet and hands were getting warm, and that in a short time she would be all right; that there was no danger whatever of her dying. I kept assuring her continually that she was getting better in every way. Now, of course, I can not say for a certainty, but I firmly believe that if I had said at the time something like this: "Well, Bessie, I am sorry that you have to die and leave us, but you will be better off. We hope to meet you in heaven. Good-by," I believe that she would have passed in her checks. I do not believe that every one who thinks that they are going to die would die, but I am referring to this particular case.

Now, of course, in a case of the last stage of consumption, or in any case where the party has but a short time to live, it is not necessary to talk in the above manner, and no one would think of doing so.

One more experience of mine and I will drop this part of the subject. Some time ago, when I had gotten a short distance from my office, I met a negro woman who was bringing her fourteen-year-old boy to me for the purpose of removing a fish bone from his throat. She said that it had been in there for two days, and that she could see it. From the symptoms she gave I little doubted it being in there. As she was good pay I told her that I would return to the office and remove the fish bone. I hurried back home, and for fear that I might not find a bone in the boy's throat, I got a small one ready in case of emergency. I had had fish for dinner that day, and I therefore had no trouble in finding one. When the boy came into the office I examined his throat carefully, but could see no bone. Tonsils were, however, irritated somewhat. I prepared a mop made of absorbent cotton, put it far enough in his throat to make him gag a little, gave the mop a turn, removed it, and as I turned around I placed a small bone onto the mop, for which I got $1.50. The woman told me afterwards that she never saw any one get along so nicely as he did after I got that bone. I wish to say, however, that I have removed quite a number of fish bones, real ones. I placed that bone on that mop for the purpose of relieving the worry from the mind of both woman and boy. That comes under the head of suggestion. I could have told her that there was no bone in the boy's throat, but charged her $1.50 treating the tonsilitis. It would not have been near so satisfactory.

Encouraging suggestion is a wonderful aid to the drugs given. If you are called to a patient suffering with intense pain, how pleasant it is to the patient for the doctor to tell him that in a few minutes he will be comparatively or entirely easy. How much better it is for you to suggest the effect that you desire the medicine to have. If you wait for them to state the effect that the medicine is having you may have to wait a long time in some cases.

(In some of the following sentences I quoted almost verbatim from a lecture on Hypnotism and Suggestion, but not being able to see the pamphlet again, I am unable to put quotation marks where required.)

Patients frequently imagine heart disease, Bright's disease or cancer, but seldom imagine mild or trivial affections. It is also easy to produce imaginary disease by suggestion, when there is some slight pain to work upon. The skillful quack, who gains the confidence of his patient, may easily make him believe that he has cancer of the liver, aneurism of the aorta, or, in fact, any disease from infantile convulsions to senile debility, and then proceed leisurely to cure him. The cure is usually as long as the patient's pocketbook. However, if the case be needed for advertising purposes, it may be remarkably short.

There are very few healthy men who do not occasionally get a pain in the chest, back or abdomen due to some trivial cause. The sickly man would pay no attention to such a thing. He is used to pain. But to the healthy man it is unusual and alarming. The assurance of a doctor may easily turn this pain into a pleurisy, nephritis or appendicitis. Here is the opportunity for the true physician to assure him that nothing is seriously the matter; provided, of course, there is not.

But what an opportunity for the quack, and how skillfully he uses it. How skillfully he disseminates his circulars and newspaper advertisements with this end in view. How many people can read the advertisement of a genius in this line without getting at least an uncomfortable feeling and a suspicion of some insidious, lurking disease? The imaginary disease is common, easy to produce, and often hard to cure. Never make any one believe he is sick in order to make a fee.

From the earliest days suggestion has been used indiscriminately, unscientifically, and in a manner that savored always so strongly of quackery as to bring it into very bad repute. While its bad repute has prevented reputable physicians from using suggestion openly, yet under other names and often unconsciously it has been used by them with great advantage. That it has done good can not be denied.

Every physician of repute knows that his reputation is a great aid to him in curing disease, and that his personal influence or personal magnetism is of great value. He is often unconscious of the fact that these things are of value because they make his patients believe in him and believe his suggestions.

The time has come when the true value of suggestion should be known, and its use placed upon a scientific and honest basis. We must know what diseases it may cure, what diseases it may aid in curing, or alleviating, and how to use it. Nearly all diseases can be benefited by suggestion, but it should seldom, if ever, be used to the exclusion of other remedies, and *never* to the exclusion of other remedies that are clearly indicated.

Now, in conclusion, I will say that all physicians use suggestion. The simple statement, "I can cure you" or "This tonic will build you up wonderfully" is suggestion. The physician who is fairly well educated in his profession, and understands the use of suggestion, will build up a practice anywhere where people get sick. It is only a matter of time.

Professional and business obligations have prevented me from getting this paper in the exact shape desired, but disconnected as it may be, if it should be the means of causing any of you to think upon the subject after going to your homes and to your daily work it will not have been written in vain.

